# Health perspectives among Halabja’s civilian survivors of sulfur mustard exposure with respiratory symptoms—A qualitative study

**DOI:** 10.1371/journal.pone.0218648

**Published:** 2019-06-21

**Authors:** Faraidoun Moradi, Mia Söderberg, Fazil Moradi, Bledar Daka, Anna-Carin Olin, Mona Lärstad

**Affiliations:** 1 Section of Occupational and Environmental Medicine, Department of Public Health and Community Medicine, Institute of Medicine, Sahlgrenska Academy, University of Gothenburg, Gothenburg, Sweden; 2 Region Västra Götaland, Närhälsan Research and Development, Primary Health Care, Gothenburg, Sweden; 3 Department of Anthropology and Philosophy, Martin Luther University Halle-Wittenberg, Halle, Germany; 4 Department of Public Health and Community Medicine/Primary Health Care, Institute of Medicine, Sahlgrenska Academy, University of Gothenburg, Gothenburg, Sweden; 5 Department of Respiratory Medicine and Allergology, Institute of Medicine, Sahlgrenska Academy, University of Gothenburg, Gothenburg, Sweden; National University of Singapore, SINGAPORE

## Abstract

**Background:**

In 1988, Halabja came under heavy chemical warfare attack using chemicals such as sulfur mustard (SM). Thousands of survivors of SM exposure in the city today live with multiple health complaints, such as severe, long-lasting respiratory symptoms; but their perceptions of health have never been adequately researched. We aimed to explore current major health concern topics in civilian survivors with long-term respiratory symptoms.

**Method:**

Sixteen subjects (f:m10:6, mean age 45.5 years (range 34–67)) were interviewed. Study participants were recruited in 2016 via a purposive sampling strategy among civilian survivors of chemical warfare in the city of Halabja in Kurdistan-Iraq. A qualitative research design was applied including semi-structured, face-to-face interviews. Data was analyzed using systematic text condensation.

**Results:**

The analysis yielded fourteen themes related to: *(1)* General health: all participants described a deterioration in physical and psychological health, following the SM exposure, foremost involving respiratory symptoms, fatigue, sleeping disorders, ocular problems, depressive symptoms, and anxiety; *(2)* Quality of life: most notably, they reported a limited family life, limited social relations, lack of work ability, and concern about their financial situation. Moreover, many lived in constant fear of a renewed attack; *(3)* access to health care services: all participants reported that they had no, or only poor, access to health care services and limited access to specialist care, and all reported lack of financial resources to obtain treatment.

**Conclusions:**

The post-exposure somatic and psychosocial effects such as respiratory symptoms of CWA are plausible contributor to poor general health and quality of life among survivors. We conclude that multidisciplinary interventions are needed to tackle the biopsychosocial complications in survivors of SM exposure to minimize further health damage in the future, as well as to promote their health-related quality of life.

## Introduction

The recent chemical attacks during civil war in Syria and Kurdistan-Iraq have shown that chemical weapons still threaten human security.[1, 2]) In 1987–1991, the Iraqi state carried out what is known as the “al-Anfal Genocide” against the Kurdish people; al-Anfal is the title of the eighth chapter of the Quran, meaning “the spoils of war.” The act of war involved use of different chemical warfare agents (CWAs), such as sulfur mustard (SM) and the nerve agents including sarin, tabun, and VX, and some sources even reported use of cyanide as well.[3–9] The Iraq Survey Group found no evidence of Iraq’s access to cyanide, but the Iraqi armed forces did have access to large amounts of SM.[10] Analyses of soil samples have confirmed Iraqi’s use of SM and sarin against civilian Kurds in Iraq.[3, 9]

The largest chemical attack was carried out on the city of Halabja in 1988, in which approximately 5,000 persons died immediately and thousands of survivors today are still suffering from long-lasting injuries.[4–6, 11] These massive civilian casualties may be explained by the fact that Iraq was using a cocktail of different CWAs, including nerve agents.[5] According to an unpublished intern report from the Ministry of Martyrs and Anfal Affairs (MMAA) within the Kurdistan Regional Government (KRG), to date about 6,000 SM survivors with multiple health complaints are registered in Kurdistan-Iraq. Whereas nerve agents have high mortality, SM has low mortality but high morbidity.[11]

Studies indicate that SM affects the respiratory and ocular systems and the skin, with both short and long-term effects. Mental health effects are also pronounced.[11–16] Delayed effects, e.g., neuropsychiatric symptoms, become obvious only in the longer term.[11] The majority of survivors of SM exposure suffer from severe, long-lasting respiratory conditions, primarily chronic bronchitis, chronic obstructive pulmonary disease (COPD), bronchiolitis obliterans, emphysema, pulmonary fibrosis, bronchiectasis, tracheal and/or bronchial deformities, and recurrent pneumonia.[11, 13, 17, 18] Many are in need of oxygen therapy and the negative impact on activities of daily living is profound.[4, 5, 19–21]

Moreover, the experience of CWAs and fear of renewed exposure to CWAs implies chronic poor health status and post-traumatic stress disorder (PTSD).[16, 22, 23] A recent study reports that SM effects on the respiratory system in particular are closely associated with psychiatric disorders.[22] A study on Iranian male war veterans revealed that SM victims with ophthalmological complications had lower health-related quality of life (HRQOL) compared to a control group.[24] Yet, knowledge regarding health and quality of life (QOL) in civilian survivors of CWAs, such as SM, is very limited. Previous studies have used the term “mustard lung” and “mustard quality of life” to refer to their condition.[25, 26] As mustard lung has different signs and symptoms from conventional lung diseases, QOL in individuals with mustard lung is different, and involves different aspects from QOL in the general population.

Survivors of the chemical attacks who report persisting health complaints are referred to and registered with the MMAA within the KRG. Being registered as a CWA-exposed patient means receiving life annuity and other social benefits. Survivors’ health status is determined according to a disability scale based on the severity of their clinical symptoms and the laboratory and radiological findings of a medical board including various specialties, e.g., pulmonologists, ophthalmologists, and dermatologists. Nevertheless, access to medical treatment is poor and there are no specific health services for survivors of SM exposure in Iraqi Kurdistan.[27]

This study aims to explore major health concerns in Halabja’s civilians with long-term respiratory symptoms after SM exposure. Increased knowledge on the topic is of high interest as it can serve as a basis for improved medical care and psychosocial interventions.

## Methods

### Study design

A qualitative study design was applied. Semi-structured interviews were performed using a previously developed interview guide (Table 1). We have followed open-ended questions specifically engaging family, marriage, social life, including work situations, and fears.

**Table 1:** Interview guide.

Could you please talk about the time Halabja was attacked with chemical gases and how you became exposed?
How do you define good health? What does good health mean to you?
How do you value your health now, compared to before you were exposed to chemical warfare agents?
How do you perceive your physical health?
How is your mental health?
Tell us about your sleep habits.
When do you feel the worst?
When do you feel better?
Why do you think this is so?
Where and how do you seek medical treatment?
How have you been met and treated by medical staff and authorities, as a patient who has been exposed to chemical warfare?
What is your family situation?
What is your work situation? How do you support yourself financially?
How many friends/family members spend time with you on a regular basis?
Do you have many friends/family members you can ask for help?
Would you like to talk about your fears?
What are your expectations?
How do you see the future; what are your dreams for the future?
Is there anything else you would like to talk about?

### Sampling

The study was performed in the city of Halabja in Iraqi Kurdistan, which has the greatest number of survivors of CWA exposure. The inclusion criteria were registration as a CWA-exposed patient, diagnosis of lung damage, and non-smoking. Exclusion criteria were severe illness requiring home-based oxygen therapy and inability to hold a 15 minute conversation at least.

### Recruitment

The first author (F.M.) identified potential participants from the patient records at the MMAA. Two non-governmental organizations, the Halabja Chemical Victims’ Society and Halabja Glory Organization, facilitated the recruitment of participants. Potential participants were invited by telephone. Those who were interested in participating received information about the study. Due to cultural considerations, the participants gave their informed consent verbally, because they clearly preferred this in advance of written informed consent. In the local context, signing documents is associated with the bureaucracy of authoritarian states and therefor written consent was viewed with distrust. Verbal informed consent was obtained by standardized questions about voluntary participation, the right to withdraw without any consequence and confidentiality and ensuring anonymity.

The Ethical Committee of College of Medicine at University of Sulaimani in Kurdistan-Iraq and the MMAA Bureau of Sulaimani approved the study including the verbal consent. The study was further approved by the Regional Ethical Review Committee in Gothenburg, Sweden.

Data was obtained through face-to-face interviews held in Sorani Kurdish, during the first week of October 2016, by the first author (F.M.). Interviewees including the three pilot interviewees were informed about his professional background and the purpose of the research at the beginning of the interviews.

The interviews were conducted at the office of the patient association or at the participant’s home depending on the interviewees’ preference. The semi-structured interviews utilized open-ended questions to provide an opportunity for the participants to talk openly about their experiences. Three pilot interviews were conducted to ensure clarity and appropriateness. However, since they did not differ considerably from the remainder of the interviews, they were included in the study. We deemed data saturation to have been reached after 16 interviews, as repetitive information emerged in the last interviews. Interviews were audio-recorded and had a mean duration of 37.5 (range 30–45) minutes. An authorized translator transcribed the interviews verbatim and translated them into English. To ensure privacy of the participants, the interviewees were allocated an anonymous code and the name of the participants was not mentioned during the recording of the interviews. Only the first author had access to the codes. The data was further anonymized by the first author (F.M.) prior to its distribution to the other authors.

### Domains of investigation

We assessed domains related to general health, quality of life and health care with a qualitative method. We drew inspiration from the World Health Organization (WHO)’s definition of general health and QOL.[28] The quality of life in many patient groups is assessed with qualitative research methods in previous studies.[29, 30]

### Data analysis

A thematic cross-case analysis was conducted using systematic text condensation.[31] This method is recognized to highlight participants’ experiences and perspectives without elaborating on their statements at length. There are few previous studies dealing with the topic, therefore the coded categories are derived directly from the text data. The data was coded by the first author, and then discussed and refined by the research team. In advance of each step, a research team meeting was held to discuss the primary outcomes and the process forward. The procedure involved four steps: *(1)* The interviews were studied separately by each research team member to get a preliminary overview, and primary themes were formulated and discussed.[28, 32] *(2)* The first author (F.M.) started the coding process by classifying the material into meaningful units, and systematically collecting the data relevant to each code across the entire material. *(3)* The codes were condensed and citations were produced and sorted into different categories. *(4)* The descriptions and concepts were presented to the research group by the first author and further refined within the research group. During the process, we returned to the main text several times to find the most appropriate citations that would best describe the main results.

## Results

The sample consisted of 16 face-to-face interviews. Participants’ demographic variables are presented in Table 2. Sixty-nine percent of the participants had blistering at the time of the gassing, which, in addition to delayed and chronic respiratory symptoms, is a further sign of exposure to SM.[12, 27] The analysis yielded fourteen themes related to: *(1)* General health; *(2)* Quality of life; and *(3)*Access to health care services, presented in Tables 3, 4 and 5. The themes are elaborated below using representative quotations.

**Table 2:** Summary of demographic variables.

Variables	n (%) *
**Age, mean (range), yrs**	45.5 (34–67)
**Sex**	
** Female**	10 (62)
**Marital status**	
** Married**	8 (50)
** Single**	4 (25)
** Divorced**	3 (19)
** Widowed**	1 (6)
**Education**	
** Primary school or lower**	9 (56)
** High school**	4 (25)
** University**	3 (19)
**Occupation**	
** Housekeeper**	4 (25)
** Employed**	5 (31)
** Unemployed**	6 (38)
** Retired**	1 (6)
**Tobacco consumption**	
** Never-smoker**	15 (94)
** Ex-smoker**	1 (6)
Alcohol consumption	0 (0)
**Children**	
** None**	6 (38)
** One to two**	3 (19)
** Three to four**	5 (31)
** Seven or more**	3 (12)

*Unless otherwise specified, values are given as number (%).

**Table 3:** The domain of general health, with themes and subthemes.

Themes	Subthemes
Physical health	Respiratory symptoms
	Ophthalmological problems
	Dermatological problems
	Fatigue
	Sleeping disorder
Psychological health	Depressive symptoms
	Anxiety
	Irritability
	Post-traumatic stress disorder symptoms
	Suicidal ideation

**Table 4:** The domain of quality of life, with themes.

Themes
Limited family life
Concern about couple relations
Limited Social relations
Limited daily life activities.
Reduced work ability
Fear of a renewed catastrophe
Fear of a renewed catastrophe
Sensitivity to changes in the weather

**Table 5:** The domain of access to health care services, with themes.

Themes
Poor access to health care services
Poor access to specialist care
Need for sustainable care and treatment
Lack of resources for specialist care

### General health

Table 3 shows the main physical and psychological health problems related to the participants’ perception of their general health. All participants described a deterioration in health, both physically and psychologically, following CWA exposure. They felt unequivocally that the deterioration was mainly due to the effects of CWAs. Many participants described SM as something that had become permanently integrated into their bodies, with a profound negative effect on their health.

*Good health means life. We don’t have good health. We have accepted it. Before the chemical attack, I didn’t take any medicine, hadn’t been to the doctor. I had a healthy body, a beautiful body—a strong body. And now you see I have no strength even for working in my own house*.

(female interviewee (FI):6)

*I feel that this effect is genetic; let’s say it has affected the structure of our body. The chemicals, I mean, they have become part of our bodies*.

(male interviewee (MI):5)

#### Physical health

In addition to respiratory symptoms, e.g., cough, dyspnea, breathlessness, sputum production, and recurrent pneumonia, many participants reported constant and escalating problems with vision and their skin. Many suffered from triple complaints. These problems affected the participants’ daily life activities.

*My lungs are not functioning. Both of my eyes have been implanted [with lenses]. Especially those parts of my skin where I had burns in 1988 get unbearably itchy*.

(MI:3)

*I can’t sleep all night. I have to go to the hospital for medicine until the itching stops*.

(FI:10)

**Fatigue.** The majority of interviewees (14 out of 16 participants) reported problems with tiredness, both mental and physical, which could be explained by, e.g., dyspnea, but also by psychological problems.

*The times I have difficulty with breathing, I can’t work at home. Even tidying up the bed would make me out of breath*.

(FI:12)

**Sleeping disorders.** Another predominant concern was problems related to sleeping. Thirteen of the 16 participants reported that they did not sleep well or enough. This was explained largely by the respiratory symptoms, but also by nightmares, flashbacks, and itching, all underlying issues that were physical and/or psychological.

*I prefer sleeping in a sitting position at night as otherwise I get out of breath. I also get anxious. It [sleeping lying down] would make me uneasy*.

(FI:14)

#### Psychological health

Despite the fact that mental illness is often perceived as “taboo” in the region, almost all participants reported a wide range of psychiatric and psychological symptoms. The majority of interviewees described that the psychological symptoms posed a daily challenge.

**Depressive symptoms.** Almost two-thirds of participants reported depressive symptoms. Many were unable to master their feelings and cried during the interview.

Actually, I always brood. I’m often in a very bad mood. I cry all the time. I miss my children [dead and missing] all the time. [Crying.]

(MI:1)

**Irritability.** The tendency to be easily upset was reported by the majority of the participants. This was perceived as a huge challenge in the participants’ daily life, both at work and at home, with negative impacts on social relations.

*I’m in a bad mood all the time … If I’m in a crowd for a bit, I immediately get moody and want to leave*.

(FI:9)

… *My family is getting fed up with me. Sometimes the simplest thing that it is not worth getting angry about … I mean I feel I have a very bad reaction* …

(MI:6)

**Anxiety.** In addition to symptoms such as irritability and depression, feeling physical and psychological uneasiness was common among the participants. Some participants reported that they felt anxious throughout the whole body.

*I’m always brooding and worried. Now, only a street separates our house from my mother’s. I go there, but can’t stay there calmly. With the keys still in my hand, I come back home … I sit for a bit. Then I go back to my mother’s. I can’t calm down there either. So, I’d say I go back and forth ten to 15 times a day until around 11 at night. Then after that I’m afraid of [being out on] the road. … I’m so restless*.

(FI:6)

**Post-traumatic stress disorder symptoms.** The majority of the participants reported signs and symptoms of unprocessed trauma even three decades after the chemical warfare attacks. Many still reported flashbacks whenever they heard about or were reminded of the attacks. The anniversary of the chemical bombardment of Halabja is one example of a powerful reminder that triggered flashbacks.

*You would feel it through your breathlessness every day …You would remember that you have been hit by chemicals* …

(MI:5)

**Suicidal ideation.** The ongoing deterioration of physical and psychological problems made the participants feel extremely desperate. Many reported suicidal thoughts as their health continued getting worse year after year and there was no treatment. They were not enjoying life. They felt that they had been abandoned, which evoked feelings of fear and hopelessness.

*Actually, death—I’m longing for it so much you can’t imagine* …

(FI:13)

*When I am chronically sick, can’t go out, can’t mingle with people […] and you wouldn’t have a future because of your illness; I don’t see a future for myself*.

(FI:14)

### Quality of life

The main findings related to QOL are illustrated in Table 4. All participants said that their health had worsened gradually, post-exposure, and that this affected all aspects of QOL.

#### Limited family life

The majority of the participants described effects on their family life, both from the perspective of being a victim themselves, and from the perspective of being a relative of a victim. They perceived themselves as burdens to their families.

*Physically we are ill and psychologically we are uncomfortable. We can’t cope with crowds. They make us uncomfortable. Now, I am even impatient with my own family and children* …

(FI:14)

… *I feel that I become angry a lot. So it’s for a while that I’ve felt that my family is about to get fed up with me* …

(MI:4)

#### Concern about couple relations

The majority also described worry about couple relations and marriage. Notably, the majority of female participants reported that exposure to CWA and its delayed effects had made it impossible to get married.

… *Her husband is about to marry again because she was wounded in the chemical attacks*.

(MI:3)

#### Limited social relations

Many described disgust and discrimination from others, both at work and in public. They thought that this probably was based on people’s lack of knowledge and understanding about their respiratory symptoms and the delayed health effects of SM exposure. The respiratory symptoms and anxiety prevented them from socializing with friends.

*I need to go outside to cough … I get breathless. Somebody might get scared when you are coughing …. That means I don’t put myself in a situation where I can annoy other people …. Often, I get unwell at events. So I don’t go to events*.

(FI:7)

#### Limited daily life activities

The majority of the participants described reduced ability to function throughout the day, which affected activities such as driving, shopping, and housework. Female participants perceived this limitation as a major challenge in their marriage as they were expected to do the housework and take care of the family.

… *How is it possible that someone of my age [46 years] is not capable of doing housework*?

(FI:12)

#### Reduced work ability

Many reported decreased performance at work, because of both physical tiredness and irritability. Reduced work ability had a negative impact on participants’ financial status and independence. Post-exposure health complaints were also reported to be a cause for quitting their jobs or hardly being able to do required work tasks.

*I am not the healthy person that I wish to be …. It has blocked my life, concerning work and business*.

(MI:4)

*We get unwell …. We get tired quickly. We are not like normal people who are able to do any work* …

(FI:9)

#### Fear of a renewed catastrophe

Insecurity and the unstable political situation in the region made many afraid of a renewed catastrophe.

*These people (in Halabja) have really suffered and sacrificed lives … They are exhausted. They couldn’t stand a repeat attack. They couldn’t stand another catastrophe* …

(MI:3)

#### Fear of post-exposure effects

The majority of interviewees expressed fear of being disabled or dying from post-exposure effects. These worries were more obvious among younger participants and those with small children. They were worried about not being able to support their family or raise their children or of remaining alone and sick.

*I expect [death]. Those before me, most of them have died. You know it, you hear about it in the news*.

(MI:13)

*I’m afraid this illness will make me disabled. That’s the only fear in my head*.

(FI:12)

*If tomorrow I would stay in bed … When I collapse, and become an invalid, who will take care of my wife and children? … I’m sure, whether it is today or tomorrow—this is the decision of God—it will kill me certainly*.

(MI:13)

#### Sensitivity to changes in the weather

Many reported deterioration of their respiratory symptoms during wintertime, when they were easily affected by respiratory infections. Because of symptoms such as dyspnea they were unable to go out for a walk, or for a longer outing.

*During spring and let’s say winter. Also in the winter if you get a cold or… But in the spring your condition would get worse because of … Let’s say you would get out of breath. Really. I mean you would even go out many times for a change of air*.

(MI:8)

### Access to health care services

The participants’ main view of the available health care services is presented in Table 5. All participants considered access to health care a fundamental need.

#### Poor access to health care services

All the interviewees mentioned that they had no, or only very poor, access to health services, even in emergencies.

*I don’t have any treatment for emergencies. Now, when I get breathless at night, where should I go? That’s my fear*.

(MI:1)

#### Poor access to specialist care

Access to specialist care was also scarce. Many expressed that the medical professionals had little or no knowledge about their health condition and even distrusted the participants regarding their illness and symptoms. They said that their pain and symptoms were invisible and that it was hard to convince medical professionals about them.

… *If I go to the hospital, they don’t know what my illness is … They would give me some medicine that would make my illness worse* …

(MI:1)

… *I have met health staff who have said: “Oh, here she comes again. What’s going on here; we are busy with only you* …?”

(MI:5)

*No, they wouldn’t believe me … You have an issue inside my body. When it’s not obvious, what should I do*?

(MI:4)

#### Need for sustainable care and treatment

The general perception was that poor access to medical care and deficient treatment meant that they had been abandoned, and that they needed more sustainable care and treatment. All participants requested international assistance to provide accessible and sustainable health care for the survivors of CWA exposure in Halabja.

… *To take care of our pain. We request those people [the international organizations] to come to the rescue of these people [the victims] … The people of Halabja need lots of help and solidarity. Healthwise and psychologically* …

(MI:3)

#### Lack of resources for specialist care

Because of financial crises and instability in the region, all participants reported difficulty in affording to buy medicine or pay for private specialist care, which they usually sought abroad, often in the neighboring country.

*It’s almost 3 years since I received any [medicine] or visited the specialist, because of the financial crisis*.

(MI:4)

## Discussion

In this qualitative study, we investigated the health perspectives of survivors of SM exposure in Halabja who had respiratory symptoms. The participants reported severe concerns regarding their general health, QOL and access to health care, which is consistent with previous studies.[16, 17, 22] All participants experienced deterioration in physical and mental health and wellbeing, and attributed their health problems to the CWA exposure, saying that the SM had become a “part of their body” and had damaged, and was continuing to damage, their organs.

The majority of the participants suffered from sleep disturbances. These were due to their respiratory symptoms, unprocessed traumatic involuntary memory, and itching problems. Inadequate sleep can adversely affect somatic and psychological wellbeing, and reduce the ability to function throughout the day, which may have been a contributing factor to the fatigue that affected almost all of the participants.[21, 33]

Mental illnesses and neuropsychiatric problems are among the delayed and chronic effects of SM exposure.[11] To date, only a few qualitative studies have been conducted regarding these effects; however, high prevalence of psychiatric problems has been reported among Iranian survivors of SM in previous studies.[15, 22]

Our findings revealed prevalence of current depressive symptoms among almost two- thirds and suicidal ideation in well above one-third of the participants. However, there is no data on the prevalence of depression, suicidal ideation, and suicide rate in Kurdistan-Iraq. One epidemiological study reports that the lifetime prevalence of a major depressive episode among the Iraqi population is 7.4%.[34] According to a WHO report, the suicide mortality rate in Iraq in 2016 was 3.0 per 100 000.[35] The methodology used in these two studies was, however, very different from that used in the present study; and still, our results found the presence of mental disorders amongst all the survivors of SM. This may be explained by underlying health problems, especially respiratory symptoms, and, in addition, experiences of social isolation, abandonment, hopelessness, and witnessing the gradual death of other SM-exposed individuals as well as possibly experiencing synergistic effects of other CWAs, e.g., nerve agents. However, suicide is the ultimate consequence of mental illness, and in other research, the incidence of suicidal ideation is well known among COPD patients.[36] One study revealed the role of hopelessness in mediating the relation between sleeping disorders and suicidal ideation.[37]

Another notable feature of this study is that respiratory symptoms are a key factor behind the deterioration in work ability, as well as being a social stigma.[38] Furthermore, lack of public knowledge about, and understanding of, SM health complications is perceived to be the main determinant behind the participants’ gradual social isolation. Others, i.e., non-victims in society, perceive the SM health complications as transmittable and hereditary. However, most of the participants used the word “we” when describing their symptoms and concerns, which can be considered as a defense mechanism against stigmatization and which suggests a strong sense of fellowship among those who suffer from similar pain and existential concerns.

Moreover, this study reveals that almost every participant suffered from poor mental health, but no one was registered at the MMAA based on psychiatric diagnosis. In addition, the absence of a psychiatric specialist on the evaluation medical board is noteworthy and remarkable. Although the direct damage from SM involves damage to the respiratory system, eyes, and skin in the first place, the psychological and psychosocial effects on victims of exposure are very pronounced. It appears that some SM survivors may have exaggerated their physical symptoms in order to be registered and examined, and get some form of care. Some psychiatric health care is available, although access to specialized care is very limited.

More than half of the participants described symptoms such as anxiety and irritability, which is consistent with previous studies.[15, 22] Participants’ anxieties included both physical and psychological signs and symptoms, and the response of SM victims to conventional psychopharmacological drugs has been questioned.[11] The survivors felt extremely desperate to get a diagnosis as they felt something was going on within their body. This feeling impacted all aspects of their lives and resulted in low quality of life. Previous studies have revealed that, among CWA effects, neuropsychiatric symptoms become obvious very late and, furthermore, are complicated to evaluate.[11, 16] In part, these symptoms may be due to the victims’ belief that the SM had contaminated their whole body and was continuing to damage their organs; in part, they may be explained by the unprocessed and involuntary traumatic experiences and memories and also the feeling of abandonment and hopelessness. Furthermore, it is well reported that respiratory symptoms could be considered a trigger factor.[38–40] There may be additional effects due to high comorbidity between COPD and psychiatric diseases.[41, 42]

Furthermore, this study shows that the majority of participants had severe worries concerning the social consequences of exposure to SM. Stigmatization and abandonment are constitutive of how the participants experienced that the community was “fed up with” them and considered them “contaminated.” This had left them with a sense of individual insecurity and uncertainty regarding their ability to support their family and marriage, as their health deteriorated more and more. Several studies have shown that exposure to SM continues to cause biopsychosocial health damages post-exposure, in particular respiratory symptoms and mental illness.[12, 15, 17, 43, 44]

Our results show that patients had limited access to basic, adequate health care, and had difficulty affording the necessary medications or visits to specialist care, and that care needs were not adequately addressed by the KRG and the federal government of Iraq.[45] The reason for this may be the financial crisis and unstable political situation in the KRG. The lack of a coordinated and functioning health care service remains an urgent concern of the SM survivors. The fact that survivors of CWA exposure in Halabja received different doses of a cocktail of CWAs, including different nerve agents, may also explain why the survivors of SM exposure in Halabja had more severe respiratory symptoms compared to SM survivors in Iran, who received initial treatment and have access to specialist care.[5, 12] The post-exposure somatic and mental effects such as respiratory symptoms of CWA, have been a plausible contributor to a particular form of physical and psychosocial nervousness. Therefore, as well as using the terms “mustard lung” and “mustard QOL,” we have formulated the term “chemical contamination anxiety.” Chemical contamination anxiety inevitably limits engagement in family, social life, and work, and results in poor general health and quality of life. Many also remain unemployed and live a vulnerable life, in regards to social and financial circumstances.

### Strengths and limitations

To our knowledge, this is the first study that focuses on SM survivors’ major health concerns and QOL in Halabja. Another strength is the range of professions represented in the research group, ensuring an inclusive, increased the credibility of analytical approach and generating data and results of high validity. Additionally, the first author’s cultural background and Sorani Kurdish language competence promoted trust that was necessary for the in-depth interviews.

The data collection method created the opportunity for the participants to decide how to describe their experiences, and in how much detail. Hence, it is not plausible to standardize the outcomes, as is the case with questionnaire surveys.

One limitation may be that all participants had respiratory symptoms. This could limit this study’s generalizability to all survivors of SM exposure. The selection of participants may have also been influenced by the Halabja Chemical Victims’ Society and Halabja Glory Organization. However, the most severely ill subjects were excluded, and the results from different interviews were uniform.

Another limitation is that the participants came from a war-ravaged environment, meaning that the cumulative trauma may have affected the outcome. The results are based on self-reporting and it may be impossible to overcome bias. The survivors of CWA exposure in Halabja received different doses of a cocktail of CWAs including nerve agents, as opposed to only SM. Therefore, it is difficult to rule out overlapping and synergistic effects.

## Conclusion

We found that about three decades after the chemical attacks, survivors of SM exposure are still suffering from biopsychosocial complications that are plausible contributor to poor general health and quality of life. We conclude that a multidisciplinary intervention program composed of medical, psychological, rehabilitative, educational, cultural and social components is needed to tackle biopsychosocial complications of CWA exposure in SM survivors. This would also prevent, or at least minimize, health damage in the future and promote the survivors’ health-reltaed quality of life.
